# Child Development and Nutritional Status in Ecuador

**DOI:** 10.1177/2333794X18821946

**Published:** 2019-01-23

**Authors:** Lourdes Huiracocha-Tutiven, Adriana Orellana-Paucar, Victoria Abril-Ulloa, Mirian Huiracocha-Tutiven, Gicela Palacios-Santana, Stuart Blume

**Affiliations:** 1 Universidad de Cuenca, Cuenca, Azuay, Ecuador; 2 University of Amsterdam, Amsterdam, Netherlands; 3 Vrije Universiteit Amsterdam, Amsterdam, Netherlands

**Keywords:** child development, complementary feeding, infant, Ecuador, nutritional status

## Abstract

We assessed the development, nutritional status, and complementary feeding of 12- to 23-month-old children from Cuenca, Ecuador in 2013. Ecuador, an upper-middle-income country, developed a child policy in accordance with World Health Organization (WHO) guidelines. We collected cross-sectional survey data. Child development was assessed using the Integrated Management of Childhood Illness Guide–2011. The nutritional status was defined with WHO Child Growth Standards−2006. We investigated nutrient density, WHO Infant and Young Child Feeding Indicators, and nutrient supplementation intake of the complementary feeding. In all, 11.7% of children had “possible developmental delay,” stunting was identified in 29.4% of the children, and 25.3% faced overnutrition (overweight risk/overweight/obesity). The complementary feeding composition can be summarized as having adequate fat, high energy (MJ/day) and protein, and low iron and zinc. Children with “possible developmental delay” received less iron (*P* < .05) than children with normal development. Overall, 30.4% of children had minimum dietary diversity. A total of 47.7% of children received nutrient supplementation. This epidemiological profile of infants remains a challenge for Ecuador’s health programs.

## Introduction

Indicators of child development and nutritional status quantify the overall health of children younger than 2 years and reflect a country’s policies, programs, and level of development.^1^ In 2007, more than 200 million children younger than 5 years in low- and middle-income countries failed to reach their full developmental potential, meaning that these children will probably have limitations in learning, socialization, and participation.^2^ In recent decades, as a consequence of a global strategy to reduce undernutrition, there has been a declining trend in the prevalence of stunting and wasting, although they remain high in regions with struggling economies.^3,4^ On the other hand, the prevalence of overweight/obesity (OW/OB) is rapidly increasing in many countries,^5^ doubling the burden of malnutrition (the coexistence of undernutrition and OW/OB in the same population or OW/OB and micronutrient defici-ency in the same person).^6^ Undernutrition, micronutrient deficiency,^7^ and OW/OB lead to a rise in morbidity and mortality in children.^8^

Adequate nutrition during the first 2 years of life from breast milk and complementary feeding (CF) promotes optimal growth and development. The World Health Organization (WHO) and the European Society for Paediatric Gastroenterology, Hepatology and Nutrition (ESPGHAN)^9^ recommend continuing breastfeeding in children that receive CF until 2 years of age. To ensure a high density of macronutrients and micronutrients, CF must include foods with a variety of flavors and textures, including bitter vegetables, meat, and allergenic foods. There is limited scientific evidence regarding CF recommendations and practices between and within countries.^10^ In the past few decades, there has been a shift toward the increased consumption of foodstuffs that are high in fat and sugars and low in fiber and micronutrients, resulting in poor dietary diversity and low nutrient density.^5,11^ A study of the quality of CF in 46 countries from 2002-2008 using the WHO Infant and Young Child Feeding Indicators (WHO IYCF)^12^ reported inadequate nutritional practices especially in diet diversity.^13^

Policies to promote the comprehensive health of children should be based on an up-to-date epidemiological profile of development, nutritional status, and CF in children younger than 2 years and the causal relationship of these variables. Relevant biological, psychological, and sociocultural factors^1^ that influence a child’s comprehensive health include the following: genetics, epigenetics,^14^ comorbidities,^7^ psychological relationship of the parents with the child,^15^ eating behavior of the child and the family,^16^ security of and access to the food supply, unequal access to health and other social services,^17^ and levels of anxiety and stress due to natural disasters or to outbreaks of violence, war, or terrorist attacks.^18^ In response to these challenges, the WHO has recommended strategies for promoting the health of women and children.^19^

In recent decades, Ecuador has aligned itself with WHO strategies. The country’s nutritional profile changed in the period 1988-2013 when Ecuador rose to the 88th position based on the Human Development Index (HDI),^20^ and the World Bank (2013)^21^ reclassified it from the lower-middle-income countries (LMICs) group to the upper-middle-income countries (UMICs).^22^ The National Health and Nutrition Survey (ENSANUT-ECU) report^23^ showed that between 1988 and 2013, there was a decrease in the prevalence of stunting (from 33.5% to 25.3%) and in underweight status (from 12.8% to 6.4%) and an increase in OW/OB status (from 4.2% to 8.6%). Wasting remained constant at 2.4%. In addition, the report revealed the high proportion of carbohydrates and fats in the infant diet, accompanied by a low intake of protein, iron, zinc, and fiber. There are no official data series on the frequency of developmental delay in children younger than 2 years.

There is a lack of information on developmental delay, nutritional status, and CF practices among young children in the city of Cuenca.^6,23^ The only available data come from a study conducted in 2008, which indicated a prevalence of 11% for child developmental delay.^24^ The present observational study focused on the development, nutritional status, and CF evaluation of 12- to 23-month-old children in Cuenca. In addition, relational analyses were carried out between these indicators. CF was investigated through the intake, nutrient density, WHO IYCF, and nutrient supplementation intake. Findings were compared with data from Latin America and the Caribbean (LAC) and from low-, lower-middle-, and upper-middle-income countries (LICs, LMICs, and UMICs, respectively),^25^ while food intake and nutrient density were compared with WHO 2002 data^10^ or Faber et al 2016 data.^11^ In this way, the similarities and differences between the profiles of Cuencano children and children from elsewhere in the world can be observed.

## Methods

### Study Population and Study Participants

A cross-sectional study of all children aged 12 to 23 months who attended the “Healthy Child Program” at 18 Public Health Centers (MSP-HC) in urban Cuenca was conducted between January and June 2013. Cuenca is located in the Sierra Region of Ecuador, and according to the 2010 census, of its population, 1.44% are indigent and 10.8% are poor; 4.9% of the population older than 15 years is illiterate, 40.9% has access to the internet, 9.6% of people with disabilities attend special education establishments, while 70% of households receive all the basic public services.^26^

The population sample was calculated with a frequency of 7.3% chronic undernutrition in Cuenca,^27^ 3% inference error, 95% confidence, and 20% losses (n = 214 children). The exclusion criteria were acute or chronic illnesses or disabilities. Although a randomization procedure was established, application of the exclusion criteria resulted in a sequential composition of the sample. A total of 214 cases were collected, respecting stratification by centers and sex. All children received iron and vitamin A supplementation in MSP-HC according to the “Norms and Protocols for Micronutrient Supplementation.”^28^

### Data Collection

The clinical evaluation and data collection were carried out by a group of health professionals: pediatricians (for verification of exclusion criteria, demographic data, and anthropometry), psychologists and early-childhood teachers (for assessing child development), and nutritionists (24-hour dietary recall).

#### Demographic Data

Surveys collected demographic data including age, sex, family residence (urban and peri-urban), family type (single-parent family: one parent plus children; or nuclear: both parents plus children), family caregiver (the person who mainly provides the child with daily care: mother or other including father, grandparents, uncle, aunt, siblings), emigration (whether father or mother or both have lived outside the country for more than a year), whether the mother had completed secondary education and, if so, when.^29^

#### Child Development

Child development was assessed using the Integrated Management of Childhood Illness (IMCI) 2011 procedure table-chart B. “No” meant normal while “possible developmental delay” indicated the absence of one or more of the skills appropriate to the child’s previous developmental age.^30^

#### Anthropometric and Nutritional Status

Anthropometric measures of weight and length were taken following WHO protocols^31^ using previously calibrated SECA instruments. Nutritional status was categorized by the WHO Child Growth Standards 2006 using weight for age *Z*-score (WAZ), length for age *Z*-score (LAZ), and body mass index (BMI) for age *Z*-score. The following definitions were used: underweight WAZ < −2SD; stunting LAZ < −2SD; wasting BMI for age *Z*-score < −2SD; overweight risk between +1SD and +2SD; overweight between +2SD and +3SD; obesity >+3SD; normal when all indicators were within −2SD and +2SD; overnutrition: overweight risk, overweight or obese.

#### Complementary Feeding

CF was determined through a 24-hour dietary recall interview using a standardized dietary kit that allowed the caregiver to specify the type and amount of food consumed by the child. The following parameters were determined: (*a*) food intake: uploaded to Nutrimid software (version 2012), which reports calories, macronutrients in grams (carbohydrates, fats, proteins, fiber) and micronutrients in milligrams (iron, zinc, calcium); (*b*) density: amount of each nutrient per 100 kcal or 416 kJ^11^; and (*c*) WHO IYCF were assessed following the methodology proposed by WHO. The questions were translated and validated in Spanish, and some questions were adapted to include the food products available in Cuenca. Four child age–specific indicators were investigated: (*a*) continued breastfeeding (CBF) (proportion of children 12-23 months of age who received breast milk during the previous day); (*b*) bottle-feeding (proportion of children fed with a bottle); (*c*) minimum dietary diversity (MDD) (proportion of children who received foods from 4 or more food groups); and (*d*) minimum meal frequency (MMF) (proportion of breastfed and nonbreastfed children who received solid, semi-solid or soft foods 3 or more times per day).^12^ Additionally, nutrient supplementation (whether or not the child had ingested a product generated in a biotechnological process with macro- or micronutrients) and the intake of commercial infant foods (infant formula, cereals, and baby foods) were recorded. No maternal milk or nutrient supplementation was quantified because of the difficulty in accurately evaluating the quantity and the quality of the nutrients.

### Statistical Analysis

Data were analyzed using SPSS software, version 21. Normality was assessed by asymmetry, kurtosis and QQ plot graphs. Mean and standard deviation (SD) were determined for “normal” variables and median and quartiles for “nonnormal” variables. The Student *t* test (normal) and Mann-Whitney *U* (nonparametric) were applied to search for significant differences (*P* < .05) in sex, stunting, overnutrition, or development. For qualitative demographic variables, prevalence percentages with CI = 95% were obtained. The chi-square (χ^2^) test in the bivariate analysis was derived by means of the contingency tables with WHO IYCF, nutrient supplementation, stunting, overnutrition, and development. The Cramer *V* statistic was used if χ^2^ was *P* < .05 in cells that had an expected frequency less than 5, measuring the relationship between variables as strong (approximately 1 and *P* < .05) or weak (approximately 0 and *P* > .05). Multiple linear regression models and multivariate logistic regression models were used to evaluate the strength of the association between a dependent variable and multiple independent variables, but no significant association was found. These types of models were useful to find confusing variables such as children’s age.

### Ethical Considerations

The Ethics Committee of the Faculty of Medical Sciences of the University of Cuenca, Ecuador approved this study with number 1811-M. Written consent was obtained from the children’s parent. The study was carried out in accordance with the ethical principles established by the Declaration of Helsinki. In addition, reports on the children’s health status were provided to the parents as well as to the physicians and health centers to ensure care after evaluation.

## Results

### Demographic Characteristics and Child Development

Table 1 summarizes the characteristics of the study population (n = 214) compared with the country data from Ecuador and LAC. Most children lived in urban areas where nuclear families are common. The mother was the main caregiver, and half of the mothers had completed secondary education. “Possible developmental delay” was identified in 11.7% of the children, and they were referred for interdisciplinary evaluation. No significant relationships were found between demographic characteristics and the following variables: stunting, underweight, wasting, overweight risk, OW/OB, and developmental delay.

**Table 1.** Demographic Characteristics and Possible Developmental Delay of 214 Children 12 to 23 Months Old in Cuenca.*.

Characteristics	% (95% CI)	Ecuador (%)	Latin America and the Caribbean (%)
Sex (Children)			
Males	49.1 (42.0-56.0)	51^a^	—
Females	50.9 (44.0-58.0)	49^a^	—
Residence			
Urban area	88.3 (84.0-93.0)	68^b^	79^b^
Peri-urban	11.7 (7.0-16.0)		
Family caregiver			
Mother	82.2 (77.0-87.0)	94^c^	90^d^
Other**	17.8 (13.0-23.0)		
Mother with complete secondary education			
Yes	51.9 (45.0-59.0)	36.6^e^	49.8^e^
No	48.1 (41.0-55.0)		
Family type			
Nuclear family	55.6 (49.0-62.0)	50.0^c^	41.1^d^
Single-parent family	44.4 (38.0-51.0)		
Parent emigration			
Yes	3.7 (1.0-6.0)	8.3^e^	5.3^e^
No	96.3 (94.0-99.0)		
Developmental delay			
Possible	11.7 (7.0-16.0)	30.0^f^	18.0^g^
No	88.3 (84.0-93.0)		

* Data were obtained from: ^a^INEC (2010),^26 b^UNICEF (2014),^61 c^Observatorio de los Derechos de la Niñez y Adolescencia (2010),^62 d^CEPAL (2007),^29 e^PNUD (2013),^63 f^Developmental delay, Handal et al (2007),^37 g^Developmental delay, Black et al (2016).^2^

** Other: father or grandparents or uncle or aunt or siblings.

### Anthropometry and Nutritional Status

Table 2 shows 7% of the children were underweight, 0.5% were wasting, 29.4% were stunted, 20.1% had a risk of being OW, 5.1% were OW, and 0.9% were OB. In children with developmental delay, 28% presented with stunting, 12% with overnutrition, and 8% with underweight, and no wasting was observed. There were significant differences in anthropometric measures regarding the weight and length of boys and girls (*P* < .05). Table 3 compares these data with the Ecuadorian data, the averages from LAC and with the highest and lowest prevalence’s found throughout the rest of the world.

**Table 2.** Anthropometry, Nutritional Status by Sex, Development, and Breastfeeding Status of 214 Children 12 to 23 Months Old.

Indicator	Total = 214	Boys = 105	Girls = 109	*P*	Developmental Delay		*P*
					Possible = 25	No = 189	
Age, months, mean (SD)	16.5 (3.5)	16.7 (3.5)	16.3 (3.5)	.33^a^	16.7 (3.2)	16.5 (3.5)	.74^a^
Weight, kg, mean (SD)	9.8 (1.25)	10.1 (1.26)	9.6 (1.21)	.00^a^*	11.2 (0.8)	10.9 (0.9)	.19^a^
Length, cm, mean (SD)	76.5 (4.58)	77.2 (4.50)	75.7 (4.58)	.02^a^*	81.1 (3.9)	80.1 (4.4)	.27^a^
BMI, kg/m^2^, mean (SD)	16.8 (1.62)	16.9 (1.72)	16.7 (1.53)	.45^a^	16.5 (1.4)	16.8 (1.7)	.52^a^
Length for age, n (%)							
Stunting	63 (29.4)	34 (32.4)	29 (26.6)	.22^b^	7 (28.0)	56 (29.6)	.54^b^
Normal	151 (70.6)	71 (67.6)	80 (73.4)		18 (72.0)	133 (70.4)	
Weight for age, n (%)							
Underweight	15 (7.0)	10 (9.5)	5 (4.6)	.13^b^	2 (8.0)	13 (6.9)	.54^b^
Normal	199 (93.0)	95 (90.5)	104 (95.4)		23 (92.0)	176 (93.1)	
BMI for age, n (%)							
Wasting	1 (0.5)	0 (0.0)	1 (0.9)	.84^b^	0 (0.0)	1 (0.5)	.05^b^
Risk overweight	43 (20.1)	23 (21.9)	20 (18.3)		2 (8.0)	41 (21.7)	
Overweight	11 (5.1)	6 (5.7)	5 (4.6)		1 (4.0)	10 (5.3)	
Obesity	2 (0.9)	0 (0.0)	2 (1.8)		0 (0.0)	2 (1.1)	
Normal	157 (73.4)	76 (72.4)	81 (74.3)		22 (88.0)	135 (71.4)	

Abbreviation: BMI, body mass index.

a Student’s *t* test for mean differences.

b Pearson χ^2^ test on contingency tables.

* Statistical significance *P* < .05.

**Table 3.** Nutritional Status of Children in Cuenca Compared With Children in Latin America/Caribbean and the Rest of the World.*

Nutritional Status	Cuenca (Huiracocha, 2013)	Ecuador, 2012^a^	Latin America and Caribbean, 2008-2012^b^	Worldwide: 2008-2012^b,c^	
				Higher Prevalence	Lower Prevalence
Lower length (stunting) (LAZ< −2SD)	29.4%	25.3%	11.0%	Afganistan^1^: 59% Burundi^1^: 58% Timor Leste^2^: 58% Yemen^2^: 58%	USA^4^: 3% Belarus^3^: 4% Singapore^4^: 4% Jamaica^3^: 5%
Lower weight (underweight) (WAZ< −2SD)	7%	6.4%	3.0%	Timor Leste^2^: 45% India^2^: 43% Yemen^2^: 43% Bangladesh^1^: 36%	Belarus^3^: 1% USA^4^: 1% Georgia^2^: 1% Former Yugoslav Republic of Macedonia^3^: 1%
Emaciation (wasting) (BMI for age< −2SD)	0.5%	2.4%	1.0%	South Sudan^2^: 23% India^2^: 20% Timor Leste^2^: 19% Niger^1^: 18%	USA^4^: 0% Turkey^3^: 1% Swazilandia^2^: 1%
Overweight and Obesity (BMI for age> +2SD)	6.0%	8.6%	7.0%	Albania^3^: 22% Lybia^1^: 22% Egypt^2^: 21% Georgia^2^: 20%	Kazakhstan^3^: 1% Oman^4^: 2% India^2^: 2% Bangladesh^1^: 2%
Risk of overweight (BMI for age between +1SD and +2SD)	21%	25.7%	—		

Abbreviations: LAZ, lenght for age Z-score; WAZ, weight for age Z-score; BMI, body mass index.

* Data were obtained from ^a^Freire et al,^23 b^UNICEF 2014^61^: Averages from 2008 to 2012 using UNICEF and WHO worldwide data statistical models, ^c^The World Bank 2013.^22^ Country classification: ^1^low income, ^2^lower middle income, ^3^upper middle income, ^4^high income.

### Complementary Feeding: Nutrient Density and WHO IYCF

Children in this study consumed more energy and protein but less fiber, iron, and zinc in their CF compared with WHO 2002 data^10^ and Faber et al data (2016)^11^ (first column). Children with “possible developmental delay” received less iron (*P* < .05) than children with normal development (Table 4).

**Table 4.** Nutrient Density (Amount per 416 kJ) of the Complementary Feeding Practice of 214 Children 12 to 23 Months Old in Cuenca.^†^.

Nutrients	WHO^a^ 2002	Total = 214	Stunting		*P* ^c^	Overnutrition		*P* ^c^	Developmental Delay		*P* ^c^
			Yes = 63	No = 151		Yes = 56	No = 158		Possible = 25	No = 189	
		Median (Interquartile Range)	Median (Interquartile Range)	Median (Interquartile Range)		Median (Interquartile Range)	Median (Interquartile Range)		Median (Interquartile Range)	Median (Interquartile Range)	
Energy (MJ/day)	2.29	3.87 (3.15, 4.72)	3.76 (3.06, 4.41)	3.99 (3.15, 4.81)	.14	3.87 (3.15 4.59)	3.86 (3.13, 4.82)	.69	3.49 (2.84, 4.78)	3.91 (3.23, 4.73)	.20
Energy (MJ/kg/body weight/day)	0.34	0.38 (0.32, 0.47)	0.39 (0.33, 0.48)	0.38 (0.32, 0.47)	.23	0.38 (0.31, 0.46)	0.38 (0.32, 0.48)	.34	0.34 (0.29, 0.48)	0.38 (0.39, 0.47)	.26
Macronutrient/416 kJ											
Protein (g)	0.9	3.61 (3.01, 3.61)	3.60 (3.09, 4.10)	3.62 (2.92, 4.17)	.86	3.67 (3.13, 4.23)	3.6 (2.96, 4.12)	.25	3.37 (2.97, 3.78)	3.66 (3.01, 4.22)	.09
Fat (g)	2.77 (2.34, 3.36)^b^	3.09 (2.59, 3.53)	2.98 (2.51, 3.36)	3.17 (2.61, 3.54)	.26	3.09 (2.58, 3.46)	3.11 (2.60, 3.56)	.60	2.94 (2.24, 3.42)	3.12 (2.61, 3.54)	.25
Carbohydrates (g)	15.27 (13.99, 16.00)^b^	14.32, (12.90, 15.70)	14.67 (13.58, 15.64)	14.21 (12.70, 15.74)	.29	14.14 (13.08, 15.95)	14.38 (12.80, 15.58)	.84	14.85 (13.25, 17.28)	14.29 (12.90, 15.51)	.17
Fiber (g)	1.40 (1.16, 1.62)^b^	0.81 (0.54, 1.10)	0.81 (0.55, 1.12)	0.81 (0.52, 1.08)	.74	0.77 (0.56, 1.02)	0.85 (0.55, 1.11)	.83	0.94 (0.51, 1.37)	0.80 (0.53, 1.06)	.22
Micronutrient/416 kJ											
Calcium (mg)	63	50.79 (30.65, 78.28)	46.94 (29.70, 75.15)	52.51 (31.29, 81.16)	.25	59.62 (32.58, 80.60)	49.30 (30.09, 75.34)	.19	51.24 (30.69, 86.24)	50.53 (30.33, 76.72)	.42
Iron (mg)	1.0	0.49 (0.33, 0.71)	0.53 (0.37, 0.74)	0.48 (0.29, 0.70)	.27	0.56 (0.33, 0.77)	0.48 (0.33, 0.70)	.34	0.42 (0.21, 0.59)	0.50 (0.35, 0.74)	.04*
Zinc (mg)	0.6	0.18 (0.12, 0.27)	0.18 (0.13, 0.32)	0.19 (0.11, 0.26)	.53	0.20 (0.11, 0.27)	0.17 (0.12, 0.27)	.85	0.15 (0.11, 0.29)	0.19 (0.12, 0.27)	.86

† Data were obtained from: ^a^WHO (2002),^10 b^Faber et al,^11 c^Mann-Whitney *U* test for mean differences.

* Statistical significance *P* < .05.

Table 5 indicates that 61.2% of the children were on CBF, 67.3% of the children were on bottle feeding (bottle feeding was based on “coladas”: carbohydrates and sugary drinks), 99.5% ate with MMF, 30.4% had MDD because they mainly ate grains, roots, and tubers (99.1%) and dairy products (74.8%), but their diets were poor in other fruit and vegetables (52.3%), vitamin A–rich fruits and vegetables (32.7%), eggs (16.8%), legumes and nuts (3.7%), and fresh foods (10.7%). More than half (52.3%) of children took nutrient supplementation. None of the children ingested commercial infant food.

**Table 5.** WHO Indicators of the Complementary Feeding Practice of 214 Children 12 to 23 Months Old in Cuenca.

Indicators Feeding Practices		Stunting		*P* ^c^	Overnutrition		*P* ^c^	Developmental Delay		*P* ^a^
	Total = 214	Yes = 63	No = 151		Yes = 56	No = 158		Possible = 25	No = 189	
	n (%)	n (%)	n (%)		n (%)	n (%)		n (%)	n (%)	
Continued breastfeeding	131 (61.2)	41 (65.1)	90 (59.6)	.27	31 (55.4)	100 (63.3)	.34	13 (52.0)	118 (62.4)	.21
Bottle feeding^b^	144 (67.3)	42 (66.7)	102 (67.5)	.51	39 (69.6)	105 (66.5)	.39	14 (56.0)	130 (68.8)	.14
Minimum meal frequency	213 (99.5)	62 (98.4)	151 (100)	.29	56 (100)	157 (99.4)	.73	24 (96.0)	189 (100)	.17
Minimum dietary diversity	65 (30.4)	21 (33.3)	44 (29.1)	.32	16 (28.6)	49 (31.0)	.44	3 (12.0)	62 (32.8)	.04*
Dairy products^c^	160 (74.8)	46 (73.0)	114 (75.5)	.41	42 (75.0)	118 (74.7)	.06	15 (60.0)	145 (76.7)	.06
Fresh foods	23 (10.7)	7 (11.1)	16 (10.6)	.54	5 (8.9)	18 (11.4)	.80	4 (16.0)	19 (10.1)	0.32
Eggs	36 (16.8)	14 (22.2)	22 (14.6)	.23	3 (5.4)	33 (20.9)	.00*	2 (8.0)	34 (18.0)	.26
Vitamin A–rich fruits/vegetables	70 (32.7)	22 (34.9)	48 (31.8)	.38	23 (41.1)	47 (29.7)	.14	4 (16.0)	66 (34.9)	.07
Grains, roots, tubers	212 (99.1)	63 (100)	149 (98.7)	1.00	56 (100)	156 (98.7)	1.00	25 (100.0)	187 (98.9)	.78
Legumes and nuts	8 (3.7)	3 (4.8)	5 (3.3)	.69	3 (5.4)	5 (3.2)	.43	1 (4.0)	7 (3.7)	1.00
Other fruits, vegetables	112 (52.3)	35 (55.6)	77 (51.0)	.55	28 (50.0)	84 (53.2)	.76	8 (32.0)	104 (55.0)	.03*
Nutrient supplementation	112 (52.3)	27 (42.9)	85 (56.3)	.98	28 (50.0)	74 (46.8)	.75	10 (40.0)	102 (54.0)	.21
Commercial infant foods	00 (00.0)									

a χ^2^ test in contingency tables.

b Food mostly taken in their bottles was “coladas” (carbohydrates and sugary drinks).

c No one took formula milk.

* Statistical significance *P* < .05.

**Figure 1.**
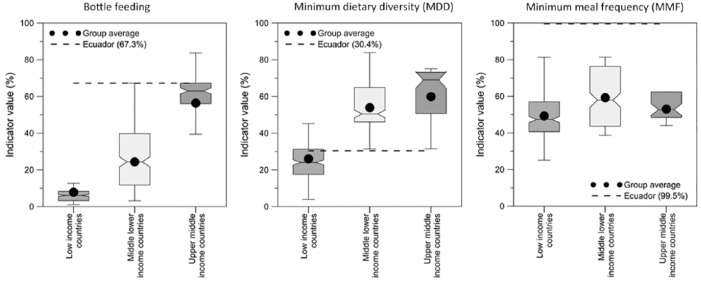
World Health Organization indicators complementary feeding practice of children in Cuenca compared with the values of other countries. Figure shows distribution and average of the percent value of the indicator for bottle feeding, minimum dietary diversity (MDD), and minimum meal frequency (MMF) for, respectively, Ecuador (dotted line), the low-income, middle-lower-income, and upper-middle-income countries.^25^

Figure 1 presents 3 subplots for bottle feeding, MDD, and MMF. In each plot, the values of the indicator derived in this research for Cuencano children (dotted line) are compared with the average indicator values for LICs, LMICs, and UMICs.^25^ The values of the bottle feeding and MMF indicators for Cuencano children are higher than in all the countries. The value of Cuenca’s MDD indicator is similar to that of the LICs.

### Relationship Between Nutritional Status, Possible Developmental Delay, and CF

Table 5 shows significant statistical relationships (*P* < .05) between the following indicators and feeding practices: (*a*) children with overnutrition consumed fewer eggs and (*b*) children with possible developmental delay had less MDD and ate fewer fruits and vegetables.

## Discussion

Demographic characteristics of the children in this study are broadly similar to the latest data reported for Ecuador and LAC (Table 1), although the percentage of mothers with completed secondary education is higher and the percentage of emigration is lower. This study found that the prevalence of “possible developmental delay” in children younger than 2 years was 11.7%, corresponding to the rate found for Cuenca in the 2008 survey.^24^ This result is difficult to compare with other investigations because of differences either in the studied populations or in the guidelines and methodologies.^32,33^ The published prevalence of child developmental delay values range from 1% to 3%,^34^ from 7.1% to 18.0%, or up to 66.0%.^35,36^ The highest rates are reported for children living in extreme poverty and in at-risk populations such as children with low birth weight, prematurity, or perinatal asphyxia. The prevalence of developmental delay, 30% in Ecuador^37^ and 18%^2^ in LAC, is typical for poor children and stunted growth.

Child development refers to the acquisition of the motor, cognitive, and social skills that enable people to carry out age-appropriate activities of daily living; to acquire academic skills; to participate in the community; and to deal with the problems they encounter. Although these skills have a genetic and biological basis, research shows that the development potential of children also depends on several factors that interact with each other. These factors include health status, nutrition, security and safety, responsive caregiving, and early learning. Two other important aspects may be noted: the immediate caring environment (family and community) and the broader social, economic, political, climatic, and cultural context. All these dimensions can have an impact on the early development of the brain and, therefore on children’s development. “Health” here includes disease prevention and treatment, and health promotion. “Nutrition” refers to dietary diversity, complementary food, macro- and micronutrients, and breastfeeding. “Security and safety” entail the reduction of threats (abuse and neglect, violence), noninstitutional family care and early intervention for vulnerable children, and birth registration. “Responsive caregiving” means responsive parenting, feeding, home-visiting, parenting programs, caregiving routines, supporting emotional development, caregiver nurturance, and continuity. “Early learning” includes transition to primary school; access to quality child care and preschool; home opportunities to explore and learn from books, toys, and play materials; and home visiting. “An enabling environment” for caregiver, family, and community is based on parental education, parental physical and mental health, age at marriage, nutrition during pregnancy, antenatal care, safe delivery, birth spacing and family planning, safe and clean neighborhoods, and absence of stigma. Finally, “social, economic, political, climatic, and cultural contexts” refers to family-supportive governance, stable governance, employment, security, housing, gender parity, the absence of extreme climatic conditions, and political commitment. When there is poverty, not all of these factors are necessarily present. However, poverty and adverse experiences in childhood have a long-term physiological and epigenetic effect on brain development and cognition.^2^ In Ecuador as a whole, poverty due to unsatisfied basic needs (UBN) (lack in the home either of housing quality, access to education or basic services or economic capacity, or overcrowding) was estimated at 38.7% in 2013^38^ and 31.8% in 2017.^39^ Multidimensional poverty (deprivation of a third of 12 indicators that relate to health, work, education, housing, and healthy environment rights) in the year 2013 at the national level was 38.7%^38^ while in 2017 it was 34.6%.^39^ For this reason, when interpreting the prevalence of 11.7% of “possible developmental delay” in the children of Cuenca, it is essential that poverty-related factors be considered.

The high percentages of both undernutrition (stunting) and overnutrition (Table 2) affirm the presence of the double burden of malnutrition. The percentages of stunting (29.4%) and underweight (7%) are comparable to national values but are almost double those of LAC (Table 3). Malnutrition is one of the dimensions to evaluate the level of development of a country, it can be deduced that the causes of the duplication of the values of stunting and underweight are due to the following. First, Ecuador with its HDI of 0.730 in 2013 was placed in the penultimate place of the 10 countries of Latin America and the Caribbean considered as Very High Human Development and High Human Development, after Argentina, Chile, Uruguay, Panama, Cuba, Costa Rica, Venezuela, Mexico, Brazil, and Peru and above Colombia. Perhaps it is therefore unsurprising that the prevalence of stunting and underweight found this study, and the data from Ecuador as a whole, were closer to values for countries in the Medium and Low Human Development group, including Bolivia, Nicaragua, Honduras, and Haiti.^40^ Second, although Ecuador, in common with all LAC countries, reduced the prevalence of poverty according to income, this decrease was slower than in the other LAC countries. Moreover, the percentage of people moving from the middle class to the vulnerable class (16%), was one of the highest among the 12 LAC countries with the best performing economies.^41^ Ecuador did not reach the level of development of other LAC countries because (as of 2013) it failed on a number of relevant dimensions. Ecuador ranks second in South America according to infant mortality rate (19.1 per 1000 live births) and children under-5 (22.5 per 1000 live births). Only 40.1% of women older than 25 years had at least some secondary education, 2.5% of the labor force had tertiary education (university), while 51.2% of employees were in vulnerable employment. In 2012, the country ranked third among countries with the highest rates of deaths due to tuberculosis (2.7 per 100 000 people) and suicide rate for women (5.2 per 100 000 women). It ranked second in terms of prevalence of violence against women (46.3%).^42^ Finally, although Ecuador aligned itself with the Millennium Development Goals and WHO strategies, as of 2013 it had failed sufficiently to expand coverage of social protection systems for poor or vulnerable people.^41^

This study also reveals that a significant percentage (21%) of children is at risk for future OW/OB. A study of obese children younger than 2 years found that 25% of them were already overweight at 3 months of age.^43^ In Latin America, more than 20% of the population was OW/OB in 4 countries, and the average rate of obesity was 4.6% in 17 countries.^44^ The increasing prevalence of overnutrition in recent years is believed to be due to the child’s genetics (birth weight), the parents’ weight,^45^ and a western lifestyle that leads to a caloric imbalance due to higher food intake and limited physical activity.^46^ The Baltimore study concludes that an increased weight for length (WFL) during the first 2 years of life increases the risk of obesity at the age of 3 years and that Latino children have a higher incidence of obesity than non-Latinos.^47^ Research in the United States indicates that the prevalence of OW/OB in children aged 0 to 2 years slightly declined from 2003-2004 (9.1%) to 2011-2012 (8.1%).^48^ Ecuador has been moving toward globalization, urbanization, with changes in occupational structures, causing the population to acquire food patterns that adapt to new lifestyles.^6,23^ Families, even the poor, consume processed or easy to prepare foods with high energy content due to the high presence of carbohydrates, sugar, salt, and fats.^5,6,8^ In Ecuador, the prevalence of obesity in adults (62.8%), the incorrect feeding of pregnant women could mark an epigenetic pattern so that children are also OW/OB.^49,50^ The risk of OW/OB increases if the inadequate diet is exacerbated by the fact that children have restrictions on moving in reduced physical spaces in the rooms or apartments where families live, or because children are held by their caregivers so they do not cry or do not hurt.^23,46^

There are studies that demonstrate the relationship between undernutrition and developmental delay^35^ while others show that developmental delay is due to the sum of biological, psychological and sociocultural factors.^1,3,51^ While cross-sectional studies do not establish cause-effect relationships, they do point to associations that merit further research. So, although the data from this study do not reveal a causal relationship between nutritional status and child development, we believe the study does suggest where future research could usefully focus.

Various recommendations for Dietary Reference Intakes (DRIs) for children have been published,^52^ but in this study, the results were compared with nutrient density values published by the WHO in 2002^10^ and by Faber et al.^11^ It was found that the CF of Cuencano children were low density (less fiber, iron, and zinc) because few of them had MDD because children mainly ate grains, roots, tubers, dairy products, but their diets were poor in fruit, vegetables, legumes, nuts and fresh foods. These findings are worrying because toddlers need healthy CF with high nutrient density and dietary diversity because they have high energy needs and eat small amounts. Children with “possible developmental delays” ingest less iron than children with normal development, perhaps because their CF is less diverse (Tables 4 and 5). This low diversity of the CF and reduced iron intake may be because the children with developmental delay due to a neurodevelopment disorder may have problems sucking, swallowing, or chewing, may demonstrate difficult feeding behavior and food selectivity, and may ingest less meat and other foods rich in iron. Moreover, iron deficiency impairs the metabolic processes of neurotransmitters in the brain, causing developmental delays in children.^51,53^

Children with overnutrition consume fewer eggs than the others (Table 5). The quality of MDD in CF of the Cuencano children is similar to LICs, but the amount of bottle feeding (with carbohydrates and sugary drinks) is higher than in all the countries (Figure 1). It is likely that this CF pattern is due to the fact that Ecuador’s socioeconomic profile is more similar to that of a LICs country than a UMIC. For all children investigated, the CF characteristics are not of high quality, which may lead to malnutrition (under and overnutrition) in the future, negatively affecting children’s growth and development.^32,54^

The CF pattern of the children found in this research is consistent with the feeding practices of Ecuadorian children reported by ENSANUT^23^, and found in other research.^55,56^ This suggests that this pattern is due to cultural practices, to historic socioeconomic influence on the production and distribution of food in different population groups,^57^ and finally due to the messages that health personnel give to the mothers. Thus, despite the availability of a variety of foods, many families prefer to give their children carbohydrates (in the form of grains, roots, and tubers cooked with water or milk, but always with sugar), while they consume little meat because they view meat as for rich people. According to the research of Waters et al,^56^ although eggs are readily available in Ecuador, and their nutritional value recognized, Ecuadorians eat few eggs. This too seems to have roots in local culture. First, the egg is reserved as a gift that is offered only to honored visitors, to healers, religious authorities, and godparents. Second, the egg is credited with healing properties. It is used to cure children of diseases caused by spells. Third, people consider that eggs raise cholesterol, and cause allergic reactions or digestion problems. Fourth, there is an economic consideration. The cost of an egg for a child is generally affordable, but when it comes to satisfying the needs of the whole family, eggs are more expensive than other foods.

The positive findings were that most of the children were CBF, achieved MMF, and did not consume commercial infant foods, and almost half of the children received nutrient supplementation from their caregiver, even if they received micronutrients from MSP-HC (Tables 3-5).

No association was found between nutritional status and CBF, MMD, or MMF. This is similar to research findings in Cambodia with children aged 6-23 months,^58^ while the Euro-Growth study found that among European children aged 12 to 24 months, there was only a weak relationship between increased weight/length and breast milk/CF consumption.^59^ However, studies in Senegal^60^ Bangladesh, Ethiopia, India, and Zambia^13^ did find an association between linear growth and WHO IYCF.

The limitations of this study were that the mothers of the children attending MSP-HC received periodic nutrition workshops. It is, therefore, possible that they concealed information about inappropriate practices in CF. The mother’s weight and height, important parameters for establishing correlations that would enable better identification of problems, were not recorded.

The results of this research indicate that Ecuadorian policies and programs, even though they have succeeded in decreasing wasting and underweight in children, have not been able to control stunting, overnutrition, or the quality of CF. Furthermore, periodic records of child development are lacking, which makes it difficult to assess the impact of public policy.

## References

[1] Walker SP Wachs TD Grantham-McGregor S et al Inequality in early childhood: risk and protective factors for early child development. Lancet. 2011;378:1325-1238. 10.1016/S0140-6736(11)60555-2 21944375

[2] Black MM Walker SP Fernald LCH et al; Lancet Early Childhood Development Series Steering Committee. Early childhood development coming of age: science through the life course. Lancet. 2017;389:77-90. 27717614 10.1016/S0140-6736(16)31389-7 PMC5884058

[3] de Onis M Blössner M Borghi E Frongillo EA Morris R. Estimates of global prevalence of childhood underweight in 1990 and 2015. JAMA. 2004;291:2600-2606. 15173151 10.1001/jama.291.21.2600

[4] de Onis M Dewey KG Borghi E et al The World Health Organization’s global target for reducing childhood stunting by 2025: rationale and proposed actions. Matern Child Nutr. 2013;9(suppl 2):6-26. 10.1111/mcn.12075 PMC6860845 24074315

[5] Cai W. Nutritional challenges for children in societies in transition. Curr Opin Clin Nutr Metab Care. 2014;17:278-284. 24531043 10.1097/MCO.0000000000000042

[6] Freire WB Silva-Jaramillo KM Ramírez-Luzuriaga MJ Belmont P Waters WF. The double burden of undernutrition and excess body weight in Ecuador. Am J Clin Nutr. 2014;100:1636S-1643S. 25411306 10.3945/ajcn.114.083766

[7] Rice AL Sacco L Hyder A Black RE. Malnutrition as an underlying cause of childhood deaths associated with infectious diseases in developing countries. Bull World Health Organ. 2000;78:1207-1221. 11100616 PMC2560622

[8] Chopra M Galbrainth S Damton-Hill I. A global response to a global problem: the epidemic of overnutrition. Bull World Health Organ. 2002;80:952-958. 12571723 PMC2567699

[9] Fewtrell M Bronsky J Campoy C et al Complementary feeding: a position paper by the European Society for Paediatric Gastroenterology, Hepatology, and Nutrition (ESPGHAN) Committee on Nutrition. J Pediatr Gastroenterol Nutr. 2017;64:119-132. 28027215 10.1097/MPG.0000000000001454

[10] Dewey KG Brown KH. Update on technical issues concerning complementary feeding of young children in developing countries and implications for intervention programs. Food Nutr Bull. 2003;24:5-28. 12664525 10.1177/156482650302400102

[11] Faber M Laubscher R Berti C. Poor dietary diversity and low nutrient density of the complementary diet for 6- to 24-month-old children in urban and rural KwaZulu-Natal, South Africa. Matern Child Nutr. 2016;12:528-545. 25138429 10.1111/mcn.12146 PMC6860079

[12] WHO; UNICEF; USAID; AED; UCDAVIS; IFPRI. Indicator for Assessing Infant and Young Child Feeding Practices. Part 2: Measurement. Geneva, Switzerland: World Health Organization; 2010.

[13] Jones AD Ickes SB Smith LE et al World Health Organization infant and young child feeding indicators and their associations with child anthropometry: a synthesis of recent findings. Matern Child Nutr. 2014;10:1-17. 23945347 10.1111/mcn.12070 PMC6860255

[14] Wang J WU Z Li D et al Nutrition, epigenetics, and metabolic syndrome. Antioxid Redox Signal. 2012;17:282-301. 22044276 10.1089/ars.2011.4381 PMC3353821

[15] Tryphonopoulos PD Letourneau N Ditommaso E. Attachment and caregiver-infant interaction: a review of observational-assessment tools. Infant Ment Health J. 2014;35:642-656. 25798513 10.1002/imhj.21461

[16] Savage JS Fisher JO Birch LL. Parental influence on eating behavior: conception to adolescence. J Law Med Ethics. 2007;35:22-34. 17341215 10.1111/j.1748-720X.2007.00111.x PMC2531152

[17] Fernandez ID Himes JH de Onis M. Prevalence of nutritional wasting in populations: building explanatory models using secondary data. Bull World Health Organ. 2002;80:282-291. 12075364 PMC2567762

[18] Programa de Naciones Unidas para el Desarrollo (PNUD). Informe sobre Desarrollo Humano. Sostener el progreso humano 2014: Reducir vulnerabilidades y construir resiliencia. New York, NY: PNUD; 2014.

[19] Ki-moon B Global strategy for women’s and children’s health. https://www.who.int/pmnch/topics/maternal/20100914_gswch_en.pdf. Published September 2010. Accessed December 14, 2018.

[20] United Nations Development Programme. Human Development Report 2015—Work for Human Development. New York, NY: United Nations Development Programme; 2015.

[21] World Bank. World Development Indicators 2013. Washington, DC: World Bank; 2013.

[22] World Bank Group. Ecuador. http://data.worldbank.org/country/Ecuador. Accessed December 14, 2018.

[23] Freire WB Ramirez MJ Belmont P et al Resumen ejecutivo. Tomo I. Encuesta Nacional de Salud y Nutrición. ENSANUT-ECU 2011-2013. https://www.paho.org/ecu/index.php?option=com_docman&view=download&category_slug=vigilancia-sanitaria-y-atencion-de-las-enfermedades&alias=452-encuesta-nacional-de-salud-y-nutricion&Itemid=599. Accessed December 14, 2018.

[24] Huiracocha ML Robalino G Huiracocha M García JL Pazán C Angulo A. Retrasos del desarrollo psicomotriz en niños y niñas urbanos de 0 a 5 años de edad: Estudio de caso en la zona urbana de Cuenca, Ecuador. Maskana. 2012;3:13-27.

[25] WHO; UNICEF; USAID; AED; UCDAVIS; IFPRI. Indicators for Assessing Infant and Young Child Feeding Practices. Part 3. Country Profiles. New York, NY: World Health Organization; 2010.

[26] Instituto Nacional de Estadísticas y Censos (INEC). Resultados de Censo 2010 en población y vivienda en el Ecuador. Quito, Ecuador: INEC; 2010 http://www.inec.gob.ec/cpv/descargables/fasciculo_nacional_final.pdf. Accessed May 11, 2015.

[27] Burgos S Calero C Molina A et al Mapa de la Desnutrición Crónica en el Ecuador. Quito, Ecuador: Ministerio de Coordinación de Desarrollo social del Ecuador, Programa Mundial de Alimentos; 2010.

[28] Ministerio de Salud Pública del Ecuador (MSP), Programa Mundial de Alimentos (WFP). Normas, Protocolos y Consejeria para la suplementación con micronutrientes. Quito, Ecuador: MSP; 2011.

[29] Comisión Económica para América Latina y el Caribe (CEPAL). Familias y políticas públicas en América Latina: una historia de desencuentros. Santiago, Chile: CEPAL; 2007.

[30] Figueiras AC Neves de Souza IC Rios VG Benguigui Y. Manual para la vigilancia del desarrollo infantil (0-6 años) en el contexto AIEPI. Segunda Edición. Washington, DC: Organización Panamericana de la Salud; 2011.

[31] Organización Mundial de la Salud. Midiendo el crecimiento de un niño. Curso de capacitación sobre la evaluación del crecimiento del niño. Geneva, Switzerland: Organización Mundial de la Salud; 2007 http://www.who.int/childgrowth/training/b_midiendo.pdf. Accessed June 9, 2012.

[32] Szajewska H. The role of meta-analysis in the evaluation of the effects of early nutrition on mental and motor development in children. Am J Clin Nutr. 2011;94(6 suppl):1889S-1895S. 21525192 10.3945/ajcn.110.000653

[33] Perkins J Rim R Krishna A Mcgovern M Aguayo V Subramanian S. Understanding the association between stunting and child development in low- and middle-income countries: next steps for research and intervention. Soc Sci Med. 2017;193:101-109. 29028557 10.1016/j.socscimed.2017.09.039

[34] Moeschler JB Shevell M; American Academy of Pediatrics Committee on Genetics. Clinical genetic evaluation of the child with mental retardation or developmental delays. Pediatrics. 2006;117:2304-2316. 16740881 10.1542/peds.2006-1006

[35] Sachdeva S Amir A Alam S Khan Z Khalique N Ansari MA. Global developmental delay and its determinants among urban infants and toddlers: a cross sectional study. Indian J Pediatr. 2010;77:975-980. 20734165 10.1007/s12098-010-0151-9

[36] Skovgaard AM. Mental health problems and psychopathology in infancy and early childhood. An epidemiological study. Dan Med Bul. 2010;57:B4193. 21040689

[37] Handal AJ Lozoff B Breilh J Harlow SD. Sociodemographic and nutritional correlates of neurobehavioral development: a study of young children in a rural region. Rev Panam Salud Publica. 2007;21:292-300. 17697482 10.1590/s1020-49892007000400004

[38] Instituto Nacional de Estadísticas y Censos (INEC). Presentación de resultados Encuesta de Condiciones de Vida (ECV) 2013- 2014. http://www.ecuadorencifras.gob.ec/documentos/web-inec/ECV/ECV_2015/. Accessed December 14, 2018.

[39] Instituto Nacional de Estadísticas y Censos. Reporte de pobreza y desigualdad. http://www.ecuadorencifras.gob.ec/documentos/web-inec/POBREZA/2017/Diciembre/Reporte%20pobreza%20y%20desigualdad%20_dic17.pdf. Published December 2017. Accessed April 20, 2018.

[40] United Nations Development Programme. Human Development Report 2013: The Rise of the South: Human Progress in a Diverse World. New York, NY: United Nations Development Programme; 2013.

[41] United Nations Development Programme. Regional Human Development Report for Latin America and Caribbean—Multidimensional Progress: Well-Being Beyond Income. New York, NY: United Nations Development Programme; 2016.

[42] United Nations Development Programme. Human development report 2015 statistical annex. http://hdr.undp.org/sites/default/files/hdr_2015_statistical_annex.pdf. Accessed October 10, 2018.

[43] Hodges EA Johnson SL Hughes SO Hopkinson JM Butte NF Fisher JO. Development of the responsiveness to child feeding cues scale. Appetite. 2013;65:210-219. 23419965 10.1016/j.appet.2013.02.010 PMC3995412

[44] Amigo H. Obesidad en el niño en América Latina: situación, criterios de diagnóstico y desafíos. Cad Saúde Pública. 2003;19(suppl 1):S163-S170. 12886446 10.1590/s0102-311x2003000700017

[45] Domínguez-Vásquez P Olivares S Santos JL. Eating behavior and childhood obesity: family influences [in Spanish]. Arch Latinoam Nutr. 2008;58:249-255. 19137987

[46] Kuzik N Carson V. The association between physical activity, sedentary behavior, sleep, and body mass index *Z*-scores in different settings among toddlers and preschoolers. BMC Pediatr. 2016;16:100. 27439395 10.1186/s12887-016-0642-6 PMC4972189

[47] Polk S Thornton RJ Caulfield L Muñoz A. Rapid infant weight gain and early childhood obesity in low-income Latinos and non-Latinos. Public Health Nutr. 2016;19:1777-1784. 27231870 10.1017/S1368980015003201 PMC10270812

[48] Ogden CL Carroll MD Kit BK Flegal KM. Prevalence of childhood and adult obesity in the United States, 2011-2012. JAMA. 2014;311:806-814. 24570244 10.1001/jama.2014.732 PMC4770258

[49] Weigel MM Armijos RX Racines M Cevallos W Castro N. Association of household food insecurity with the mental and physical health of low-income urban Ecuadorian women with children. J Environ Public Health. 2016;2016:5256084. 27752266 10.1155/2016/5256084 PMC5056290

[50] Lecoutre S Petrus P Ryden M Breton C. Transgenerational epigenetic mechanisms in adipose tissue development. Trends Endocrinol Metab. 2018;29:675-685. 30104112 10.1016/j.tem.2018.07.004

[51] Yakoob MY Lo CW. Nutrition (micronutrients) in child growth and development: a systematic review on current evidence, recommendations and opportunities for further research. J Dev Behav Pediatr. 2017;38:665-679. 28746059 10.1097/DBP.0000000000000482

[52] Hermoso M Tabacchi G Iglesia-Altaba I et al The nutritional requirements of infants. Towards EU alignment of reference values: the EURRECA network. Matern Child Nutr. 2010;6(suppl 2):55-83. 22296251 10.1111/j.1740-8709.2010.00262.x PMC6860534

[53] Sidrak S Yoong T Woolfenden S. Iron deficiency in children with global developmental delay and autism spectrum disorder. J Paediatr Child Health. 2014;50:356-361. 24372984 10.1111/jpc.12483

[54] Yehuda S Rabinovitz S Mostofsky DI. Nutritional deficiencies in learning and cognition. J Pediatr Gastroenterol Nutr. 2006;43(suppl 3):S22-S25. 17204975 10.1097/01.mpg.0000255847.77034.a4

[55] Roche ML Gyorkos TW Blouin B Marquis GS Sarsoza J Kuhnlein HV. Infant and young child feeding practices and stunting in two highland provinces in Ecuador. Matern Child Nutr. 2017;13:1-15. 10.1111/mcn.12324 PMC6866177 27265847

[56] Waters WF Gallegos CA Karp C Lutter C Stewart C Lannotti L. Cracking the egg potential: traditional knowledge, attitudes, and practices in a food-based nutrition intervention in highland Ecuador. Food Nutr Bull. 2018;39:206-218. 29558837 10.1177/0379572118763182

[57] Paez DA. Pensando una epidemiología para la alimentación: Una genealogía de los estudios nutricionales en Ecuador. Salud Colectiva, 2018;14:607-622. http://revistas.unla.edu.ar/saludcolectiva/article/view/1538. Accessed May 10, 2016. 30517566 10.18294/sc.2018.1538

[58] Reinbott A Kuchenbecker J Herrmann J et al A child feeding index is superior to WHO IYCF indicators in explaining length-for-age *Z*-scores of young children in rural Cambodia. Paediatr Int Child Health. 2015;35:124-34. 10.1179/2046905514Y.0000000155 PMC4462840 25226288

[59] Haschke F Ziegler EE Grathwohl D. Fast growth of infants of overweight mothers: can it be slowed down? Ann Nutr Metab. 2014;64(suppl 1):19-24. 25059802 10.1159/000360505

[60] Bork K Cames C Barigou S Cournil A Diallo A. A summary index of feeding practices is positively associated with height-for-age, but only marginally with linear growth, in rural Senegalese infants and toddlers. J Nutr. 2012;142:1116-1122. 22535757 10.3945/jn.112.157602

[61] Fondos de las Naciones Unidas para la Infancia (UNICEF). El Estado Mundial de la Infancia 2014 en cifras. Todos los niños y niñas cuentan. Revelando las disparidades para impulsar los derechos de la niñez. New York, NY: UNICEF; 2014 https://www.unicef.org/spanish/publications/index_71829.html. Accessed May 10, 2016.

[62] Observatorio de los Derechos de la Niñez y Adolescencia. Los niños y niñas del Ecuador a inicios del siglo XXI. Quito, 2010 https://www.unicef.org/ecuador/Encuesta_nacional_NNA_siglo_XXI_2_Parte1.pdf. Accessed May 2, 2016.

[63] Programa de las Naciones Unidas para el Desarrollo (PNUD). Informe sobre Desarrollo Humano 2013. El ascenso del Sur: Progreso humano en un mundo diverso. New York, NY: PNUD; 2013 http://www.undp.org/content/dam/venezuela/docs/undp_ve_IDH_2013.pdf. Accessed June 11, 2016.

